# Excessive daytime sleepiness and metabolic syndrome in men with obstructive sleep apnea: a large cross-sectional study

**DOI:** 10.18632/oncotarget.19113

**Published:** 2017-07-08

**Authors:** Yiqun Fu, Huajun Xu, Yunyan Xia, Yingjun Qian, Xinyi Li, Jianyin Zou, Yuyu Wang, Lili Meng, Xulan Tang, Huaming Zhu, Huiqun Zhou, Kaiming Su, Dongzhen Yu, Hongliang Yi, Jian Guan, Shankai Yin

**Affiliations:** ^1^ Department of Otolaryngology Head and Neck Surgery & Center of Sleep Medicine, Shanghai Jiao Tong University Affiliated Sixth People’s Hospital, Shanghai, China; ^2^ Otolaryngological Institute of Shanghai Jiao Tong University, Shanghai, China; ^3^ Clinical Research Center, Shanghai Jiao Tong University School of Medicine, Shanghai, China

**Keywords:** excessive daytime sleepiness, metabolic syndrome, obesity, obstructive sleep apnea

## Abstract

**Purpose:**

Excessive daytime sleepiness is a common symptom in obstructive sleep apnea (OSA). Previous studies have showed that excessive daytime sleepiness is associated with some individual components of metabolic syndrome. We performed a large cross-sectional study to explore the relationship between excessive daytime sleepiness and metabolic syndrome in male OSA patients.

**Methods:**

A total of 2241 suspected male OSA patients were consecutively recruited from 2007 to 2013. Subjective daytime sleepiness was assessed using the Epworth sleepiness scale. Anthropometric, metabolic, and polysomnographic parameters were measured. Metabolic score was used to evaluate the severity of metabolic syndrome.

**Results:**

Among the male OSA patients, most metabolic parameters varied by excessive daytime sleepiness. In the severe group, male OSA patients with excessive daytime sleepiness were more obese, with higher blood pressure, more severe insulin resistance and dyslipidemia than non-sleepy patients. Patients with metabolic syndrome also had a higher prevalence of excessive daytime sleepiness and scored higher on the Epworth sleepiness scale. Excessive daytime sleepiness was independently associated with an increased risk of metabolic syndrome (odds ratio =1.242, 95% confidence interval: 1.019-1.512). No substantial interaction was observed between excessive daytime sleepiness and OSA/ obesity.

**Conclusions:**

Excessive daytime sleepiness was related to metabolic disorders and independently associated with an increased risk of metabolic syndrome in men with OSA. Excessive daytime sleepiness should be taken into consideration for OSA patients, as it may be a simple and useful clinical indicator for evaluating the risk of metabolic syndrome.

## INTRODUCTION

Obstructive sleep apnea (OSA) is estimated to occur in 9-24% of adults, with a two- to three-fold greater risk for men than women [1, 2]. Excessive daytime sleepiness (EDS) is an important feature of OSA [2], and its prevalence is in the range 2.5-26.1% according to several studies in many different countries [3-10]. According to an Asian multi-ethnic study, the prevalence of EDS in the Chinese population is 9.4% [5]. Patients with EDS may be unable to remain awake or sufficiently alert to accomplish activities of daily life safely and successfully, which affects their cognitive function, contributes to decreased productivity and increased risk of occupational accidents, and impairs quality of life [11-15].

OSA is independently associated with metabolic syndrome (MetS), a combination of excess abdominal obesity, dyslipidemia, hypertension, hyperglycemia, and insulin resistance [16]. The pathogenesis of OSA (i.e., intermittent hypoxia and sleep fragmentation) may directly contribute to increases in risk factors that comprise MetS via pathological changes including sympathetic activation, oxidative stress, and systemic inflammation [17, 18]. Some studies have reported that EDS is associated with some individual components of MetS, i.e., obesity, insulin resistance and hypertension [19-21]. However, most of them only assessed the relationships between EDS and individual components of MetS, rather than including all five components and considering MetS in its entirety. In addition, it has been argued that EDS and MetS may share a common pathogenic mechanism (i.e., hypoxia), or that EDS may contribute to MetS by itself [19, 22, 23].

An important feature of OSA is that it occurs mostly in males. EDS is also male-dominated and mainly affects occupations requiring concentration and/or physical labor [24]. The impact of EDS may therefore result in greater social and economic harm in men than women. For these reasons, we carried out a large cross-sectional study to explore the relationship between EDS and MetS in men with OSA, and to investigate whether the prevalence and the severity of MetS are influenced by EDS.

## RESULTS

### Basic characteristics

A total of 2,241 males with suspected OSA (median age: 40 years (33, 50); mean body mass index [BMI]: 26.85 ± 3.66 kg/m^2^; mean Epworth sleepiness scale [ESS] score: 9.09 ± 5.80; and mean metabolic score: 2.48 ± 1.32) were included. In the OSA group, patients with EDS presented with more severe obesity indices (i.e., BMI, neck circumference [NC], waist circumference [WC] and waist-to-hip ratio [WHR], *p* < 0.001); higher blood pressure (*p* < 0.01); higher fasting glucose, insulin, and HOMA-IR levels (*p* < 0.001), more severe lipid abnormalities (i.e., higher total cholesterol [TC], triglycerides [TG], low-density lipoprotein [LDL], apolipoprotein B [apoB], and apoE, *p* < 0.001, and lower high-density lipoprotein [HDL], *p* < 0.05), and worse sleep parameters (i.e., higher apnea-hypopnea index [AHI], oxygen desaturation index [ODI], micro-arousal index [MAI], and lower lowest oxygen saturation level [LSpO_2_], *p* < 0.001) than non-EDS patients. OSA patients with EDS had a higher prevalence of MetS (62.6% *vs.* 48.0%, *p* < 0.001) and higher metabolic score (2.84 ± 1.21 *vs*. 2.43 ± 1.27; *p* < 0.001) than non-EDS patients. Additionally, the prevalence of abdominal obesity, hypertension, hypertriglyceridemia and hyperglycemia was significantly higher among OSA patients with EDS (*p* < 0.01; Supplementary Table 1).

The prevalence of EDS was 23.8% in the non-OSA group, 31.9% in the mild OSA group, 36.1% in the moderate OSA group, and 57.7% in the severe OSA group (*p* for trend < 0.01, Figure 2A). The ESS score increased as the severity of OSA increased (*p* for trend < 0.01, Figure 2B). In the non-OSA group, subjects with EDS had higher BMI, NC, and MAI than non-EDS subjects (*p* < 0.05). In the mild and moderate OSA groups, no significant differences were found between EDS and non-EDS subgroups for most variables (except TG in the mild OSA group, *p* = 0.022). However, in the severe OSA group, significant differences were observed between the EDS and non-EDS groups: patients with EDS were more obese (as evidenced by a higher BMI and WHR, and larger NC, WC, and HC, *p* < 0.01), with higher systolic and diastolic blood pressure (*p* < 0.05), more severe insulin resistance (*p* < 0.01), more critical lipid abnormalities (as evidenced by higher levels of TC, TG, LDL, apoB, and apoE, *p* < 0.01), and worse sleep parameters (higher AHI, ODI, MAI, and lower LSpO_2_, *p* < 0.001; Supplementary Table 2). In the severe OSA group, patients with EDS showed a higher prevalence of MetS (68.0% *vs.* 57.6%, *p* < 0.001) and a higher metabolic score (3.01 ± 1.16 *vs.* 2.67 ± 1.19, *p* < 0.001) than patients without EDS. Moreover, the prevalence of abdominal obesity, hypertension, hypertriglyceridemia and hyperglycemia was significantly higher among severe OSA patients with EDS than those without EDS (*p* < 0.05, Table 1). In addition, subjects with both OSA and MetS showed a higher prevalence of EDS (55.5% *vs.* 40.7%, *p* < 0.01) and scored higher on the ESS than OSA patients without MetS (10.51 ± 5.74 *vs*. 8.61 ± 5.50, p < 0.01).

**Figure 1:**
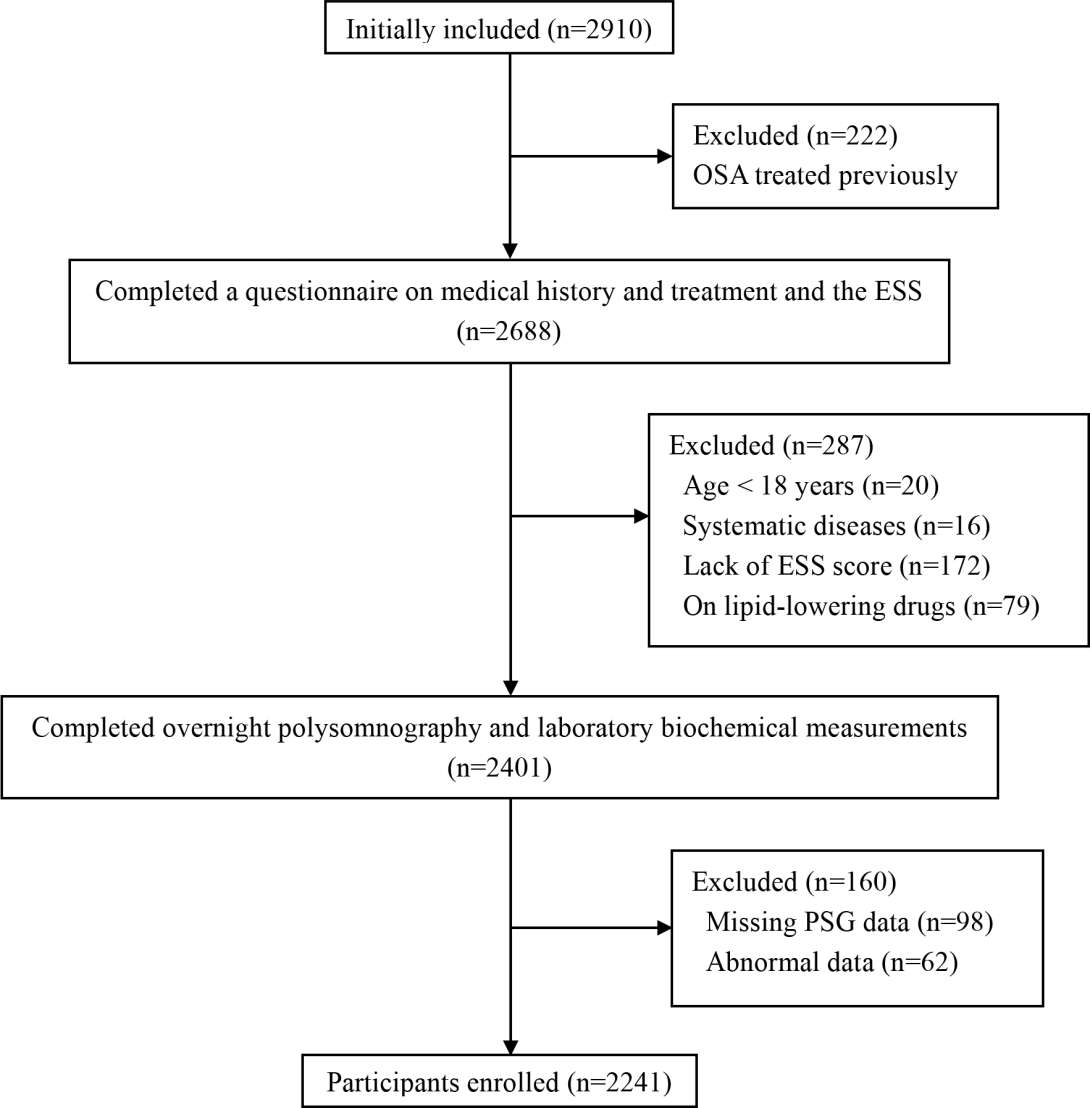
Enrollment flow chart for the study population The Shanghai Sleep Health Study cohort included 2910 males who were enrolled in our sleep center for snoring and/or EDS from January 2007 to July 2013. In total, 2241 patients met the inclusion criteria and were enrolled in this study. OSA, obstructive sleep apnea; ESS, Epworth Sleepiness Scale; PSG, polysomnography.

**Figure 2:**
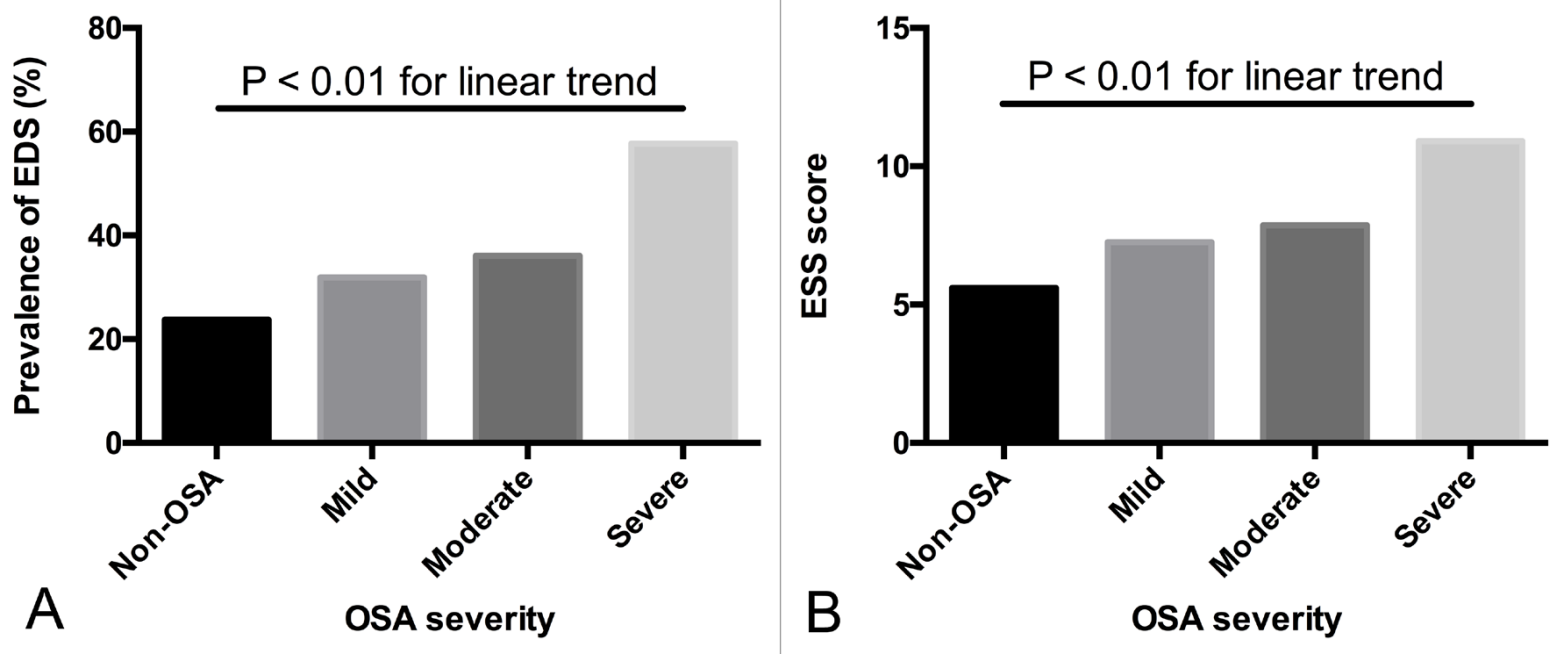
Prevalence of EDS and ESS score in different OSA categories **A.** The prevalence of EDS increased as the severity of OSA increased (*p* for trend <0.01). **B.** The ESS score increased as the severity of OSA increased (*p* for trend < 0.01). The linear-by-linear association test was used for dichotomous variables and the polynomial linear trend test was used for continuous variables.

**Table 1:** Prevalence of MetS and its components among the study participants according to OSA and EDS status.

*OSA status*	*Normal*		*p*	*Mild*		*p*	*Moderate*		*p*	*Severe*		*p*
*EDS status*	Normal	EDS		Normal	EDS		Normal	EDS		Normal	EDS	
*No. patients*	240	75		250	117		225	127		510	697	
*MetS(%)*	23.3	33.3	0.084	30.4	39.3	0.091	45.8	54.3	0.123	57.6	68.0	<0.001
*Abdominal obesity(%)*	43.8	45.3	0.810	65.2	66.7	0.783	76.0	84.3	0.068	85.5	92.7	<0.001
*Hypertension(%)*	29.2	28.0	0.846	32.0	29.9	0.688	41.8	40.2	0.767	46.1	52.4	0.031
*Hypertriglyceridemia(%)*	25.8	32.0	0.295	37.2	44.4	0.186	47.6	52.8	0.349	50.0	59.5	0.001
*Low HDL(%)*	44.2	49.3	0.433	43.6	46.2	0.646	52.0	52.8	0.892	54.1	54.1	0.992
*Hyperglycemia(%)*	9.2	16.0	0.096	18.0	23.1	0.254	23.6	29.1	0.249	31.0	42.3	<0.001
*Metabolic score*	1.52±1.22	1.71±1.45	0.317	1.96±1.27	2.10±1.17	0.304	2.41±1.32	2.59±1.23	0.205	2.67±1.19	3.01±1.16	<0.001

Data are presented as the median with the interquartile range (IQR) if skewed, means ± standard deviation (SD) if normally distributed, or number (percentage) if categorical. Normally distributed variables were analyzed via the independent sample *t*-test, and skewed variables were analyzed via the Mann–Whitney *U-*test. Categorical variables were analyzed using the chi-square test or Fisher’s exact test. EDS, excessive daytime sleepiness; OSA, obstructive sleep apnea; MetS, metabolic syndrome.

We used analysis of variance (ANOVA) to evaluate the separate effects of EDS and OSA on metabolic indicators, and to evaluate the interaction effects between EDS and OSA. The results showed that OSA significantly influenced the majority of metabolic indices (i.e., BMI, NC, WC, HC, WHR, systolic and diastolic blood pressure, fasting glucose level and insulin level, HOMA-IR, TC, TG, HDL, LDL, apoB, apoE, and metabolic score, *p* < 0.001; and apoA-I, *p* = 0.031), which resulted in more severe obesity, higher blood pressure, worse insulin resistance, and dyslipidemia. EDS also significantly affected most obesity indices (i.e., BMI, *p* < 0.001; NC, WC and WHR, *p* = 0.015, 0.002 and 0.004, respectively), contributed to glycometabolic disorders (as evidenced by higher levels of fasting plasma glucose and insulin, *p* = 0.001 and 0.003, respectively; and larger HOMA-IR, *p* < 0.001), and increased TG levels (*p* < 0.001) and metabolic score (*p* = 0.001). No substantial interactions were found between EDS and OSA status in most metabolic indices, with the exception of BMI, WC, and LDL level (*p* = 0.029, 0.007 and 0.022, respectively).

### Association between EDS and MetS

**Figure 3:**
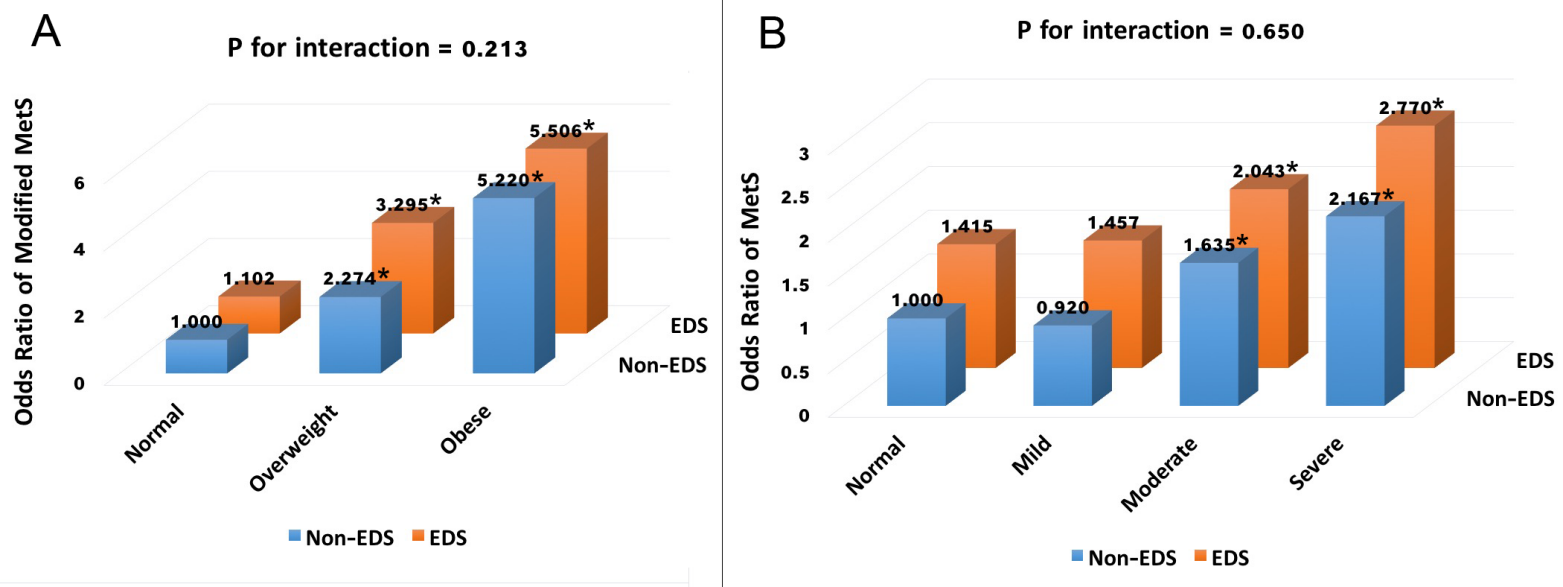
ORs for metabolic syndrome according to obesity/OSA categories and EDS status ORs according to joint classification of EDS status and obesity categories **A.** were adjusted for age, smoking status, alcohol consumption, ODI and MAI. Modified metabolic syndrome was defined as having two or more components of metabolic syndrome without abdominal obesity. ORs according to joint classification of EDS status and OSA categories **B.** were adjusted for age, BMI, smoking status and alcohol consumption. * *p* < 0.05.

Significant correlations were found between ESS score and the following components of MetS: WC, systolic blood pressure (SBP), diastolic blood pressure (DBP), fasting plasma glucose (FPG), TG, and HDL level (*p* < 0.001); and a significant positive association between ESS score and metabolic score was found (*r* = 0.224, *p* < 0.01). The polysomnographic characteristics (i.e., AHI, ODI, MAI, and LSpO_2_), smoking status, and alcohol consumption were also significantly correlated with ESS score (Supplementary Table 3). Multiple linear regression showed that ESS score was an independent predictor of metabolic score after adjusting for age, obesity, other metabolic parameters, sleep parameters, current smoking, and alcohol consumption (*β* = 0.026, adjusted *r*^*2*^ = 0.919, *p* = 0.041). In the multivariate logistic models, we assessed the associations between EDS and MetS (Table 2). EDS significantly increased the risk of MetS as a whole after adjusting for age, obesity, smoking status, and alcohol consumption (OR = 1.396, 95% CI: 1.153 - 1.698). When sleep parameters were taken into account, the association was slightly attenuated but remained significant (OR = 1.242, 95% CI: 1.019 - 1.512). To evaluate the associations between EDS and each component of MetS, we additionally controlled for the other components of MetS. In model 1 (Table 2), EDS significantly increased the risk of abdominal obesity (OR = 1.331, 95% CI: 1.000-1.770), hypertriglyceridemia (OR = 1.274, 95% CI: 1.062-1.528) and hyperglycemia (OR = 1.422, 95% CI: 1.163-1.740). When sleep parameters were taken into account, EDS was still significantly related to an increased risk of hyperglycemia significantly (OR = 1.258, 95% CI: 1.022-1.549). To determine if obesity/ OSA status could modify the association between EDS and MetS, joint classification analyses were performed. No substantial interaction was found for the risk of MetS between EDS status and obesity (*p* for interaction = 0.213, Figure 3A), or OSA status (*p* for interaction = 0.650, Figure 3B).

**Table 2:** Adjusted odds ratios for metabolic syndrome (MetS) and its components according to EDS status.

	Model 1		Model 2	
	OR	95%CI	OR	95% CI
MetS	1.396	(1.153, 1.689)	1.242	(1.019, 1.512)
Abdominal obesity	1.331	(1.000, 1.770)	1.125	(0.838, 1.511)
Hypertriglyceridemia	1.274	(1.062, 1.528)	1.189	(0.986, 1.433)
Low-HDL	0.946	(0.791, 1.130)	0.980	(0.817, 1.176)
Hyperglycemia	1.422	(1.163, 1.740)	1.258	(1.022, 1.549)
Hypertension	1.034	(0.863, 1.238)	0.958	(0.796, 1.153)

ORs for MetS were adjusted for age, BMI, WHR, smoking status and alcohol consumption in model 1, plus ODI and MAI in model 2.

## DISCUSSION

The results of this study show that among male OSA patients, most metabolic parameters were correlated with EDS. In the severe OSA group, patients with EDS had more severe metabolic disorders and a higher prevalence of MetS than non-EDS patients. Patients with MetS also had a higher prevalence of EDS and scored higher on the ESS than those without MetS. Multivariate logistic regression revealed that EDS was significantly associated with a higher risk of MetS, mainly related to an increased risk of hyperglycemia. No substantial interaction effect on MetS was observed between EDS and OSA.

This is the first study to evaluate comprehensively the relationships between EDS, MetS, and OSA with a large sample size. A positive correlation between EDS and OSA has previously been reported [13-15, 25]. Here, we also showed that the prevalence of EDS was higher in OSA patients and increased with OSA severity (Figure 2). Nocturnal hypoxemia is the cardinal feature of OSA and may be a major determinant of EDS [25-27]. The mechanisms of nocturnal hypoxemia that contribute to EDS in OSA patients are unclear. Several rodent studies reported that nocturnal hypoxemia could activate oxidative and inflammatory pathways, triggering neuronal apoptosis and neurocognitive dysfunction, ultimately leading to neural damage to regions of the brain that control wakefulness [28-30].

Obesity is associated with EDS independently of OSA [31-33], and obesity or abdominal obesity is associated with both OSA and EDS in men [34, 35]. Thus, obesity may modify the association between EDS and MetS. A previous review described that obesity is associated with abnormal circulating systemic hormones and elevated basal levels of the sympathetic nervous system, which may contribute to daytime sleepiness [36]. This study found that patients with EDS were more obese, and had a higher prevalence of abdominal obesity than non-EDS patients. Obesity affected the relationship between EDS and MetS, but only to a limited degree. Joint classification analysis suggested an independent role of EDS based on the lack of an interaction between EDS and being overweight or obese (Figure 3A). Therefore, our findings suggest that EDS contributes to metabolic abnormalities via mechanisms other than obesity.

EDS is independently associated with metabolic variables, and that the relationship is dependent on severity [4, 37]. In this study, we showed that most metabolic parameters of OSA patients with EDS, including obesity, blood pressure, fasting glucose level and lipid profiles, differed from those in non-EDS patients. Multivariate logistic regression analysis revealed that EDS significantly increased the risk of MetS. The effects of EDS upon components of MetS were more obvious in hyperglycemia after controlling for all of the other confounders. A number of studies have investigated relationships between EDS and components of MetS. Feng *et al.* found that the ESS score was positively correlated with blood pressure after controlling for potential confounders, including AHI [37]. In contrast to the effects of CPAP treatment on lowering blood pressure among OSA patients with EDS, no significant effects on blood pressure were found in OSA patients without EDS [20, 38-41]. However, it has also been reported that in non-sleepy patients, long-term CPAP treatment contributes to a small reduction in blood pressure [42, 43]. Lee *et al.* [44] found that EDS patients had higher serum levels of TC and LDL compared to non-EDS patients, while other studies showed that CPAP treatment could contribute to a reduction in cholesterol in sleepy patients [41, 45]. Barceló *et al.* [19] found that EDS is a marker of insulin resistance in OSA patients, and that CPAP could improve insulin resistance status in patients with EDS but had no effect in non-sleepy subjects. However, the European ESADA study [46] reported that EDS had no significant independent modifying influence on glycosylated hemoglobin (HbA1c) levels. While not entirely consistent, these previous studies emphasize the serious effects of EDS upon MetS and its components, and indicate that patients with EDS benefit more from CPAP treatment than non-sleepy patients.

The pathophysiological mechanisms by which EDS interacts with the components of MetS are likely to be complex and multifactorial. EDS is typically accompanied by nocturnal hypoxemia and sleep fragmentation; thus, dysregulation of the autonomic nervous system, alterations in hypothalamic-pituitary-adrenal (HPA) axis activity, and increased systemic inflammation may significantly contributed to the development of MetS [37, 47, 48].

There are a number of limitations to our study. First, we used a subjective ESS score to assess EDS rather than an objective method; however, ESS is convenient (i.e., quick and inexpensive) and is a more discriminating test of daytime sleepiness than either the maintenance of wakefulness test (MWT) or the multiple sleep latency test (MSLT) [49]. Second, the cross-sectional design does not enable us to establish a cause-and-effect relationship between EDS and MetS; hence, community-based studies are warranted to demonstrate such an association. Third, we did not perform abdominal magnetic resonance imaging to collect related data on visceral adipose tissue. Instead, we use WHR to evaluate abdominal adiposity. Last, selection bias may be significant, as we included subjects referred from our sleep center.

In summary, we used a large cohort to investigate the relationship between EDS and MetS, and found that EDS was associated with metabolic disorders and independently related to a higher risk of MetS as a whole among male OSA patients. Our study revealed a cross-sectional association, but further research is needed to determine whether a causal relationship exists between EDS and MetS. We conclude that EDS should be taken into consideration for OSA patients, as it could be a simple and useful clinical indicator for evaluating the risk of MetS. Additionally, compensating for EDS in OSA may improve metabolic dysregulation in patients.

## MATERIALS AND METHODS

### Subjects

The data reported here are from the Shanghai Sleep Health Study (SSHS) cohort. The study considered 2,910 males who were enrolled in our sleep center for snoring and/or EDS from January 2007 to July 2013. The participants were mainly from cities in southeast China, and all completed surveys for smoking habits, alcohol consumption, and medical history. Among them, 669 were excluded for the following reasons: previous treatment for OSA (*n* = 222); various systematic diseases (i.e., malignancy, chronic kidney disease, unstable cardiopulmonary disease such as congestive heart failure or intrinsic pulmonary disease) (*n* = 16); missing PSG data (*n* = 98); lack of an ESS score (*n* = 172); aged less than 18 years (*n* = 20); abnormal data (*n* = 62); and taking lipid-reducing medications prior to the study (n = 79). A total of 2,241 males were included in the analysis (Figure 1).

Written informed consent was obtained according to the guidelines outlined by the National Ethics Regulation Committee, and the study was approved by the Internal Review Board of the Institutional Ethics Committee of Shanghai Jiao Tong University Affiliated Sixth People’s Hospital.

### Anthropometric and metabolic measurements

All measurements were made using standard methods, in which the participants were dressed in lightweight clothing with bare feet. Neck circumference (NC) was measured at the level of the laryngeal prominence, waist circumference (WC) between the 12th rib and the iliac crest, and hip circumference (HC) at the level of the anterior superior iliac at the broadest circumference below the waist. The waist-to-hip ratio (WHR) was calculated as WC divided by HC. The body mass index (BMI) was calculated as body mass in kilograms divided by the square of the patient’s height in meters. A BMI in the range 18.5-22.9 kg/m^2^ was considered normal, 23-27.5 kg/m^2^ overweight, and >27.5 kg/m^2^ obese [50].

According to the guidelines of the American Society of Hypertension [51], blood pressure was measured at approximately 08:00 h, with patients in a seated position after a 5-min rest, using a mercury sphygmomanometer; readings were recorded as the mean of three measurements taken at 1-min intervals.

Fasting blood samples were taken from the antecubital vein of each patient in the morning after polysomnographic monitoring. Serum fasting glucose and serum lipid profiles were measured in the hospital laboratory using routine procedures. Serum lipid profiles included total cholesterol (TC), triglycerides (TG), high-density lipoprotein (HDL), low-density lipoprotein (LDL), apolipoprotein A-I (apoA-I), apolipoprotein B (apoB), apolipoprotein E (apoE), and lipoprotein(a) (Lpa) (Hitachi, Tokyo, Japan). An immunoradiological method was used to measure the fasting serum insulin level. Insulin sensitivity was evaluated using the homeostasis model assessment of insulin resistance (HOMA-IR). HOMA-IR was calculated as the product of the fasting insulin level (μU/mL) and the fasting glucose level (mmol/L) divided by 22.5 [52].

### Polysomnographic evaluation

Overnight polysomnography (PSG) (Alice 4 or 5, Philips Respironics, Pittsburgh, PA, USA) was performed from 22:00 h to 06:00 h according to the American Academic Sleep Medicine criteria [53], including electroencephalogram (EEG), left and right electrooculogram (EOG), genioglossus electromyogram, electrocardiogram (ECG), pulse oxygen saturation, nose and mouth airflow, thoracic-abdominal movement, and body position. The PSG variables included the apnea hypopnea index (AHI), oxygen desaturation index (ODI), lowest pulse oxygen saturation (LSpO_2_), and micro-arousal index (MAI). Apnea was defined as the complete cessation of airflow lasting for at least 10 s; hypopnea was defined as either a ≥ 50% reduction in airflow for at least 10 s or a discernible reduction in airflow accompanied by either a decrease in oxyhemoglobin saturation of ≥ 4% or arousal. The AHI is defined as the number of apnea and hypopnea events per hour during sleep. The ODI is defined as the number of episodes of ≥ 4% arterial oxyhemoglobin desaturation per hour of sleep. The MAI is defined as the number of arousals per hour of sleep. The severity of OSA was determined via AHI; i.e., < 5 events/h was considered as the absence of OSA, 5-15 mild, 15-30 moderate, and > 30 events/h severe OSA [53].

### Epworth sleepiness scale (ESS)

Daytime sleepiness was assessed using a well-validated Chinese version of the ESS questionnaire quantifying the self-reported expectation of “dozing” in a variety of situations, and included eight items [54, 55]. The questionnaire was completed by each patient on the day of admission to evaluate the subjective EDS with a four-point scale, as follows: 0, would never doze off; 1, slight chance of dozing; 2, moderate chance of dozing; and 3, high chance of dozing, under the following situations: sitting and reading; watching TV; sitting, inactive in a public place (e.g., a theater or a meeting); as a passenger in a car for one hour with no break; lying down to rest in the afternoon when circumstances permit; sitting and talking to someone; sitting quietly after lunch with no alcohol; and while stopped for a few minutes in traffic in a car. The total ESS score was in the range 0-24, and EDS was considered as an ESS score of at least 10 [54, 55].

### Metabolic score

MetS was defined according to the NCEP ATP III criteria (with the modified waist circumference criteria for Asians [56]) as the presence of at least three of the following five clinical features: WC ≥90 cm in men and ≥80cm in women; TG ≥1.70 mmol/L; HDL <1.03 mmol/L in men and <1.30 mmol/L in women; SBP ≥130 mmHg or DBP ≥85 mmHg or currently taking antihypertensive medication; and fasting glucose level ≥5.6 mmol/L or currently taking anti-diabetic medication. A metabolic score was defined as the total number of positive diagnostic criteria of MetS for each participant.

### Statistical analysis

SPSS (version 23.0, SPSS Inc., USA) was used for all statistical analyses. All data were assessed as to whether they were normally distributed prior to statistical analysis. Data are presented as the median with the interquartile range (IQR) if skewed, means ± standard deviation (SD) if normally distributed, or number of subjects *n* (%) if categorical. Normally distributed variables were analyzed via the independent sample *t*-test, and skewed variables were analyzed via the Mann-Whitney *U-*test. Categorical variables were analyzed using the chi-square test or Fisher’s exact test. ANOVA was used to compare the means of the quantitative data and to evaluate the interaction effects between EDS and OSA. Skewed data were log-transformed to obtain a normal distribution. Correlations between ESS score and anthropometric, polysomnographic, and metabolic parameters were evaluated using Spearman’s correlation test. Multiple linear regression was used to examine the determinants of metabolic score. Multivariate logistic regression analysis was used to determine whether EDS increased the risk of MetS as a whole together or each component individually. Joint classification analysis was used to evaluate the interaction effects. The statistical analysis was preceded by the use of collinearity diagnostics to eliminate possible multicollinearity among variables. A *p*-value < 0.05 was considered to indicate statistical significance.

## SUPPLEMENTARY MATERIALS TABLES









## Abbreviations

EDS: excessive daytime sleepiness
OSA: obstructive sleep apnea
MetS: metabolic syndrome
OR: odds ratio
CI: confidence interval
BMI: body mass index
NC: neck circumference
WC: waist circumference
HC: hip circumference
WHR: waist-to-hip ratio
SBP: systolic blood pressure
DBP: diastolic blood pressure
ESS: Epworth Sleepiness Scale
FPG: fasting plasma glucose
FINS: fasting insulin
HOMA-IR: homeostasis model assessment of insulin resistance
TC: total cholesterol
TG: triglyceride
HDL: high-density lipoprotein
LDL: low-density lipoprotein
apoA-I: apolipoprotein A-I
apoB: apolipoprotein B
apoE: apolipoprotein E
Lp(a): lipoprotein(a)
AHI: apnea-hypopnea index
LSpO_2_: lowest oxygen saturation
ODI: oxygen desaturation index
MAI: micro-arousal index
ANOVA: analysis of variance
CPAP: continuous positive airway pressure
HPA: hypothalamic-pituitary-adrenal
MWT: maintenance of wakefulness test
MSLT: multiple sleep latency test
SSHS: Shanghai Sleep Health Study
PSG: polysomnography
EEG: electroencephalogram
EOG: electrooculogram
ECG: electrocardiogram
NCEP ATP III: national cholesterol education program adult treatment panel III
SPSS: Statistical Product and Service Solutions
IQR: interquartile range
SD: standard deviation
