# Supplementary material for: Excessive daytime sleepiness and metabolic syndrome in men with obstructive sleep apnea: a large cross-sectional study
Source: Oncotarget. 2017 Jul 8;8(45):79693–702. doi: 10.18632/oncotarget.19113 (PMC5668082; doi:10.18632/oncotarget.19113)
Supplement: Supplementary file 1 [file oncotarget-08-79693-s001.pdf]

Excessive daytime sleepiness and metabolic syndrome in men with obstructive sleep apnea: a large cross-sectional study

SUPPLEMENTARY MATERIALS

For Supplementary Tables see in Supplementtray Files
